# Geographical Traceability of *Zanthoxylum schinifolium* Sieb. et Zucc. Using Stable Isotope and Multi-Element Fingerprinting Combined with Chemometrics

**DOI:** 10.3390/foods15061088

**Published:** 2026-03-20

**Authors:** Wei Zhang, Tingting Zeng, Tingting Fu, Yongchuan Huang, Bingjing Ji, Xia Meng, Yongyang Fan, Mingfeng Tang

**Affiliations:** 1 Institute of Agricultural Quality Standard and Testing Technology, Chongqing Academy of Agricultural Sciences, Chongqing 401329, China; 2 Agricultural Product Quality and Safety Supervision, Inspection and Testing Center, Ministry of Agriculture and Rural Affairs, Chongqing 401329, China

**Keywords:** *Zanthoxylum schinifolium* Sieb. et Zucc., geographical origin traceability, C/H/N/O stable isotope, multi-element fingerprinting

## Abstract

Accurately tracing the geographical origin of *Zanthoxylum schinifolium* Sieb. et Zucc. is important for brand authentication, quality control, and food safety assurance. In this study, the stable isotope ratios (δ^13^C, δ^15^N, δ^2^H, δ^18^O) and the contents of 20 elements were analyzed in samples from three major production regions. Significant differences (*p* < 0.05) were observed in δ^13^C, δ^2^H, δ^18^O and most elemental profiles across origins. Chemometric methods—including principal component analysis (PCA), orthogonal partial least squares-discriminant analysis (OPLS-DA), and linear discriminant analysis (LDA)—were applied to classify samples by geographical origin. OPLS-DA identified key discriminators (VIP > 1) such as Ca, δ^13^C, Mg, δ^2^H, B, δ^18^O, Cr, Ni, Na, Pb, As, Co, Se, and Zn, achieving a classification accuracy of 96.8%. LDA based on the combined isotope and element datasets showed even higher performance, with an original discrimination rate of 98.4% and a cross-validated rate of 92.8%. The results demonstrate that integrating stable isotope and multi-element fingerprints with supervised classification models provides a reliable and effective approach for verifying the geographical origin of *Zanthoxylum schinifolium*, supporting its use in traceability systems and fair trade practices.

## 1. Introduction

China is the world’s leading producer and consumer of *Zanthoxylum* spp., known as “huajiao,” with an annual output exceeding 500,000 tons supplying both domestic and international markets [1]. The two main varieties, red huajiao (*Z. bungeanum*) and green huajiao (*Z. schinifolium*), are highly valued for their distinctive aroma and numbing sensation (“ma”). Beyond its culinary applications, huajiao is also recognized in traditional medicine for its health benefits, supported by modern studies identifying bioactive compounds with various pharmacological properties [2,3]. To enhance the economic value of regional specialties, China has implemented the “Protected Geographical Indication (PGI)” system for several key huajiao production areas. However, even within the same botanical variety, huajiao from different geographical origins can exhibit variations in quality and flavor, leading to notable price differences. Therefore, establishing reliable and credible methods for authenticating geographical origin is essential for protecting brand reputation, ensuring product integrity, and maintaining fair trade in this important industry.

The authentication of an agricultural product’s provenance relies on traceability systems that decode the unique environmental imprints—often termed “fingerprints”—acquired during its growth cycle [4]. This premise is grounded in the well-established understanding that a plant’s final phenotype and chemical composition are not innate but are continuously molded by its surrounding environment. Factors such as local climate, soil geochemistry, and water sources collectively contribute to a stable and region-specific signature within the plant tissue [4]. As such, geographical traceability is the core technical support for the protection of PGI products, the maintenance of fair trade in the agricultural product market, and the protection of consumers’ legitimate rights and interests. In recent years, with the increasing attention to the authenticity and quality of regional characteristic agricultural products, geographical traceability has become a research hotpot in the field of food science and quality control [5].

The accuracy of geographical traceability is directly linked to food safety and the stability of the global food supply chain [6]. First, inaccurate traceability will lead to the influx of counterfeit and shoddy products into the market, which not only damages the brand reputation of high-quality production areas and the economic interests of smallholder farmers, but also brings significant food safety risks [7]. For example, products from non-standard planting areas with excessive heavy metals or pesticide residues may be impersonated as high-quality PGI products, endangering the health of consumers. Second, the lack of accurate traceability technology will lead to the inability to quickly locate the source of food safety incidents in the supply chain, expanding the scope of the incident and causing greater economic losses and social impact. Third, with the globalization of the food trade, accurate geographical traceability has become an important technical barrier to international trade, and reliable traceability methods are the prerequisite for Chinese characteristic agricultural products to enter the global market. Therefore, establishing a stable, accurate, and low-cost geographical traceability method is of great practical significance for ensuring food safety, maintaining the stability of the supply chain, and promoting the high-quality development of the characteristic agricultural product industry.

Various analytical techniques have been employed to verify the geographical origin of food and agricultural products. These include mass spectrometry (e.g., IRMS, ICP-MS) [8,9,10], spectroscopy (such as NIR and NMR) [11], chromatographic methods (e.g., GC, HPLC) [12,13], biochemical assays (e.g., DNA, RNA and protein analysis) [14], and sensory evaluation [15], often combined with chemometric tools. Among these, stable isotope ratio analysis and multi-element profiling have emerged as particularly effective approaches, owing to their ability to reflect environmental and agronomic influences on plant composition. These methods have been successfully applied to a wide range of products including fruits (peach, pear, and durian) [10,16,17], vegetables [18,19], herbs [20], meat [21,22], tea [23,24], wine [25,26], and aquatic foods [27]. However, most existing studies have concentrated on *Zanthoxylum bungeanum* (red pepper) using techniques such as differential pulse voltammetry [28] and hyperspectral imaging [29], with limited attention given to *Zanthoxylum schinifolium* (green pepper). To date, a comprehensive geographical traceability model integrating isotopic and multi-element signatures with multivariate statistics for *Z. schinifolium* remains to be established.

In this study, we aimed to develop a reliable discrimination model for *Zanthoxylum schinifolium* from three major production regions in China. Stable isotope ratios (δ^13^C, δ^15^N, δ^2^H, δ^18^O) and the concentrations of 20 elements were analyzed, and chemometric methods including principal component analysis (PCA), orthogonal partial least squares-discriminant analysis (OPLS-DA), and linear discriminant analysis (LDA) were applied for origin classification. The results provide a robust scientific basis for authenticating the geographical origin of *Zanthoxylum schinifolium*, supporting quality control, brand protection, and sustainable industry development.

## 2. Materials and Methods

### 2.1. Zanthoxylum schinifolium Sieb. et Zucc. Collection and Pre-Treatment

**Figure 1:**
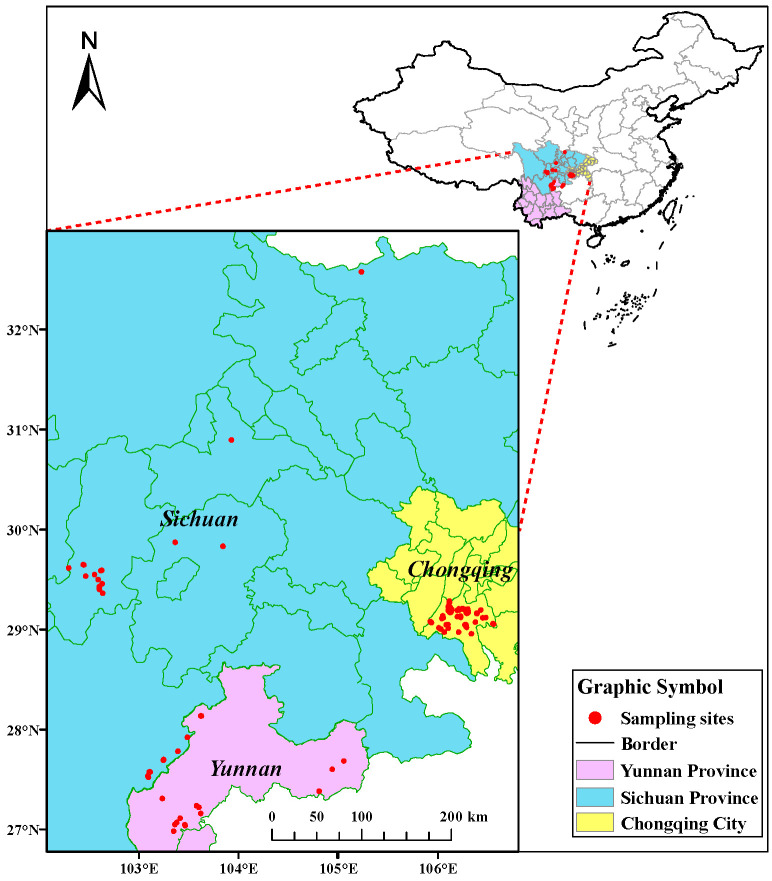
Geographical location information of *Zanthoxylum schinifolium* Sieb. et Zucc. samples from different regions in China.

In May to October 2024, 125 samples of *Zanthoxylum schinifolium* Sieb. et Zucc. were collected from three primary cultivation regions in southern China (CQ = Chongqing; SC = Sichuan Province; YN = Yunnan Province). Details regarding sampling locations (Figure 1), geographical coordinates, average altitude, and annual mean temperature are provided in Table 1. The sample sizes across the three regions were uneven (Chongqing: 85; Sichuan: 25; Yunnan: 15), reflecting deliberate design considerations: the larger Chongqing dataset was prioritized to support subsequent fine-scale analysis of producing areas within the region, while the Sichuan and Yunnan samples, though smaller, provide essential geographical context for broader comparative purposes. Although all three regions feature comparably extensive cultivation areas, this sampling strategy enables both intra-regional variability assessment in the primary study area and inter-regional comparison across major production zones. After collection, approximately 1 kg of each sample was processed by separating the peel and seeds. The material was then oven-dried at 80 °C until constant weight, finely ground with an agate mortar and pestle, and sieved through a 100-mesh screen to obtain a homogeneous powder for subsequent stable isotope and elemental analysis.

### 2.2. Stable Isotope Ratio Analysis

Stable isotope ratios (δ^13^C, δ^15^N, δ^2^H, δ^18^O) in the peel of *Zanthoxylum schinifolium* Sieb. et Zucc. were determined according to established procedures [24,30] using an elemental analyzer (Vario Pyro Cube, Elementar, Hanau, Germany) coupled to an isotope-ratio mass spectrometer (IsoPrime100, Isoprime Ltd., Manchester, UK). For δ^13^C and δ^15^N analysis, approximately 8.0 mg of sample was weighed into tin capsules. For δ^2^H and δ^18^O, about 0.5 mg was weighed into silver capsules, freeze-dried for five days, and subsequently equilibrated under laboratory conditions for three days prior to analysis.

Isotopic compositions are reported in delta notation (δ) according to Equation (1):

(1) $$\delta X=\frac{R_{sample}-R_{standard}}{R_{standard}}\times 1000‰$$

where X denotes the heavy isotope (^13^C, ^15^N, ^2^H, or ^18^O), *R_sample_* and *R_standard_* represent the heavy-to-light isotope ratios (e.g., ^13^C/^12^C, ^15^N/^14^N, ^2^H/^1^H, ^18^O/^16^O) in the sample and reference materials, respectively. Values are expressed in per mil (‰). δ^13^C is reported relative to Vienna Pee Dee Belemnite (VPDB), δ^15^N relative to atmospheric N_2_ (AIR), and δ^2^H and δ^18^O relative to Vienna Standard Mean Ocean Water (VSMOW). Calibration was performed using the following reference materials: B2155, BCR-657, USGS 64, IAEA-N-2, and USGS 40 for δ^13^C and δ^15^N; USGS 54, USGS 55, and USGS 56 for δ^2^H and δ^18^O. Analytical precision, monitored with an in-house quality-control sample, was better than 0.1‰ for δ^13^C, 0.2‰ for δ^15^N, 3.0‰ for δ^2^H, and 0.5‰ for δ^18^O.

### 2.3. Elemental Composition Analysis

The elemental analysis was performed as described in our previously published study [10], using inductively coupled plasma optical emission spectrometry (ICP-OES; Optima 8300, PerkinElmer, Waltham, MA, USA) and inductively coupled plasma mass spectrometry (ICP-MS; ELAN DRC II, PerkinElmer, Waltham, MA, USA). Prior to analysis, approximately 0.2 g of dried powdered sample was digested with 5.0 mL nitric acid (HNO_3_) at 120 °C for 3 h. Subsequently, 1.0 mL hydrogen peroxide (H_2_O_2_) was added and heating was continued for an additional hour at the same temperature. The solution was then evaporated until the volume was reduced to approximately 1.0 mL. After cooling to ambient temperature, the digestate was diluted to a final volume of 25 mL with deionized water. For quality assurance, certified reference materials (GBW10048a(GSB26a)) were processed identically in each digestion batch. Results from the reference material analyses are presented in Table S3.

### 2.4. Data Analysis

Statistical analyses were conducted using SPSS 27.0 (SPSS Inc., Chicago, IL, USA) and SIMCA 14.0 (Umetrics, Umea, Sweden). In SPSS, the Shapiro–Wilk test was initially applied to assess data normality [31], followed by a nonparametric Kruskal–Wallis test to compare differences in stable isotope ratios and elemental concentrations across geographical origins at a 95% confidence level [32]. A Kaiser–Meyer–Olkin (KMO) test [33] and stepwise linear discriminant analysis (LDA) were also carried out within the same software platform [34]. LDA is a classic supervised classification technique that maximizes inter-group variance while minimizing intra-group variance to construct a linear discriminant function for sample categorization. It is widely regarded as a benchmark method for validating geographical origin discrimination models in food traceability research [34]. Stepwise LDA was used to establish discrimination models based on the single isotope dataset, single element dataset, and combined dataset, respectively. Model performance was evaluated by the original discrimination rate and leave-one-out cross-validation (LOOCV) discrimination rate.

Multivariate modeling was performed in SIMCA. PCA is an unsupervised, unbiased method commonly used to explore data structure without prior class information [35]. PCA was performed as an exploratory visualization tool to assess natural data distribution. Diagnostic metrics, including Hotelling’s *T*^2^ and DModX, were calculated for transparency and are provided in the Supplementary Materials (Figure S1); however, no samples were excluded based on these metrics to preserve the full exploratory nature of the analysis. Orthogonal Projections to Latent Structures Discriminant Analysis (OPLS-DA), a supervised multivariate method, was then performed. OPLS-DA decomposes spectral data into components correlated with class separation and those orthogonal to it, thereby filtering out non-specific systematic variation and enhancing group differentiation [36]. This technique is commonly used for identifying discriminant variables and classifying geographical origins of agricultural products. Variable Importance in Projection (VIP) was used to screen key discriminant variables (VIP > 1). To assess model robustness and guard against overfitting, a permutation test with 200 iterations was conducted. Together, PCA, OPLS-DA, and permutation testing enabled comprehensive exploration of data structure, effective classification of samples by origin, and validation of model reliability.

## 3. Results and Discussion

### 3.1. Analysis of Stable Isotope Ratios

Table 2 presents the stable isotope composition (δ^13^C, δ^15^N, δ^2^H, and δ^18^O) of *Zanthoxylum schinifolium* Sieb. et Zucc. samples collected in 2024. For comparison across the three regions (CQ, SC, and YN), the mean ± standard error of each isotope is summarized in Table 3. A nonparametric test (Kruskal–Wallis test) revealed significant regional differences (*p* < 0.05) in δ^13^C, δ^2^H, and δ^18^O values. The δ^13^C values of all samples fell within the range of −30 to −24.5‰, aligning with previously reported δ^13^C ranges for C3 plants (−33 to −23‰) [13,32]. Across the three major production areas, the average δ^13^C values ranked as follows: YN > SC > CQ, with CQ samples showing significantly lower δ^13^C values than SC and YN (*p* < 0.05). In plants such as *Zanthoxylum schinifolium*, δ^13^C values primarily reflect isotopic fractionation during growth rather than differences in photosynthetic carbon fixation pathways. This spatial variation is mainly driven by the climatic and geographical heterogeneity of the three regions. Yunnan and Sichuan have higher altitude and stronger photosynthetically active radiation, which reduces the stomatal conductance of *Zanthoxylum schinifolium* leaves, decreases intercellular CO_2_ concentration, and thus weakens the ^13^C fractionation during ribulose-1,5-bisphosphate carboxylase/oxygenase (Rubisco)-mediated carbon fixation, resulting in higher δ^13^C values in plant tissues. In contrast, Chongqing has a lower altitude, higher relative humidity, and weaker light intensity, leading to higher stomatal conductance, higher CO_2_ concentration, and more significant ^13^C fractionation, which is the core driver of the lower δ^13^C values in CQ samples [37,38,39].

The δ^2^H values for all samples ranged from −109‰ to −24.1‰ (Table 2), with average values following the order CQ > SC > YN, and significant differences among all three regions (*p* < 0.05). Samples from CQ exhibited a significantly higher mean δ^2^H value (−72.3‰) compared to those from SC and YN (*p* < 0.05) (Table 3). The δ^2^H signature of plant tissues is predominantly inherited from the local precipitation, which is shaped by the continental effect, altitude effect, and temperature effect. Chongqing has the highest annual mean temperature (20.5 °C) and is the closest to the ocean among the three regions, so local precipitation has a higher δ^2^H value, which is absorbed by plant roots and enriched in peel tissue via xylem transportation. Yunnan has the lowest temperature (13.33 °C) and highest altitude (1653.33 m), resulting in the strongest rainout effect and the lowest δ^2^H value of precipitation and plant tissues (Table 1). A similar pattern has been reported in tobacco, where elevated δ^2^H values in certain growing regions have been linked to warmer, more tropical climatic conditions [40]. Beyond temperature, factors such as distance from the coast, irrigation water source, altitude, longitude, and humidity are also known to influence δ^2^H variation in plants [16,41].

The δ^18^O values in the analyzed *Zanthoxylum schinifolium* Sieb. et Zucc. samples ranged from 21.4‰ to 30.3‰ (Table 2). Samples from SC exhibited a significantly higher mean δ^18^O value (26.4‰) compared to those from the other regions (*p* < 0.05) (Table 3). The δ^18^O signature of plants is jointly determined by the δ^18^O of soil water, evaporative enrichment of leaf water during transpiration, and altitude. The planting areas of Sichuan are mainly distributed in the hilly areas of the Sichuan Basin, with moderate altitude, high relative humidity, and strong evaporative enrichment of ^18^O in leaf stomata, resulting in higher δ^18^O values in plant tissues. Yunnan has the highest altitude, low temperature, and weak transpiration intensity, so the δ^18^O enrichment effect is negligible, leading to the lowest δ^18^O value, consistent with the classical theory of oxygen isotope fractionation in terrestrial plants [42]. Additionally, our analysis revealed a significant statistical relationship among δ^2^H, δ^18^O, and δ^13^C values (*p* < 0.001), consistent with earlier reports that these isotopes often respond similarly to environmental drivers [43].

### 3.2. Analysis of Elemental Composition

In this study, a total of 20 elements were analyzed in *Zanthoxylum schinifolium* Sieb. et Zucc. samples from three geographical regions, comprising four macroelements (K, Ca, P, and Mg) and sixteen microelements (Na, Mn, Fe, Sr, Ba, Zn, Cu, Ni, B, Cr, Pb, Cd, Mo, As, Se, and Co) (Table 2). Among the macroelements, potassium (K) showed the highest mean content (20,045 ± 2567 mg/kg), followed by calcium (Ca, 6886 ± 2074 mg/kg), phosphorus (P, 3465 ± 560 mg/kg), and magnesium (Mg, 1897 ± 470 mg/kg). The microelements, in descending order of mean concentration across the three regions, were as follows: Na (196 ± 174 mg/kg) > Mn (65.7 ± 34.3 mg/kg) > Fe (60.9 ± 16.6 mg/kg) > Sr (26.2 ± 10.7 mg/kg) > Ba (24.0 ± 13.1 mg/kg) > Zn (21.8 ± 36.4 mg/kg) > Cu (8.72 ± 3.20 mg/kg) > Ni (6.35 ± 3.38 mg/kg) > B (6.30 ± 3.79 mg/kg) > Pb (0.757 ± 2.389 mg/kg) > Cr (0.428 ± 0.348 mg/kg) > Cd (0.279 ± 0.335 mg/kg) > Mo (0.201 ± 0.160 mg/kg) > Co (0.103 ± 0.070 mg/kg) > Se (0.0327 ± 0.016 mg/kg). Regarding variability, only K and P exhibited low variation, whereas Ca, Mg, and Fe displayed moderate variation. All remaining elements including Na, Mn, Sr, Ba, Zn, Cu, Ni, B, Cr, Pb, Cd, Mo, As, Se, and Co were highly variable.

To identify significant elemental markers for discriminating among the three production origins of *Zanthoxylum schinifolium* Sieb. et Zucc., a Kruskal–Wallis test was performed (Table 3). The results indicated that most elements differed significantly across origins, except for K, Mn, Fe, Sr, Ba, Cu, Pb, Cd, and As (*p* > 0.05). Compared with samples from other regions, CQ samples were richer in Ni, B, Cr, Cd, and Se but contained lower concentrations of Ca, P, Mg, Na, Zn, Pb, Mo, and Co. These differences likely reflect variations in growing environment factors, such as soil properties (pH and mineral composition), fertilizer use, local climate, and irrigation water characteristics [44,45]. Given these region-specific elemental patterns, we plan to apply multivariate statistical methods to develop a discrimination model for the geographical origin of *Zanthoxylum schinifolium*.

### 3.3. Multivariate Statistical Analysis

**Figure 2:**
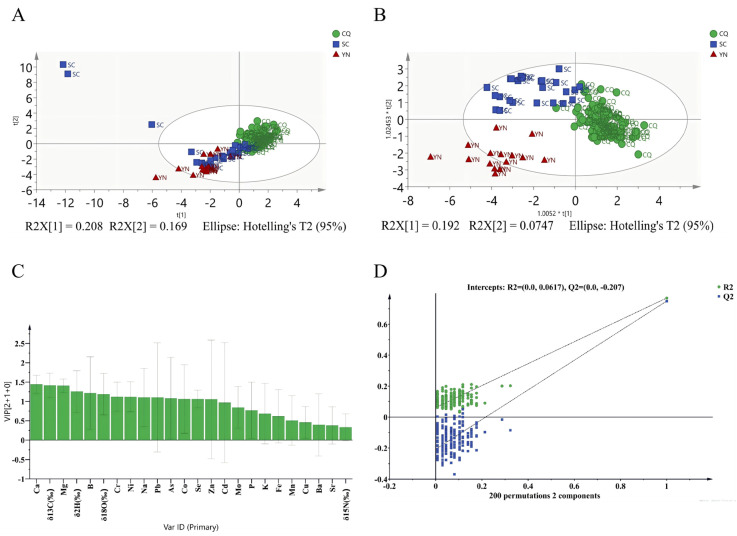
(**A**) PCA score plots and (**B**) OPLS-DA score plots of *Zanthoxylum schinifolium* Sieb. et Zucc. from three origins with the data of stable isotope and multi-element. (**C**) Variable importance in the projection (VIP) was obtained from the OPLS-DA model. (**D**) Permutation test of the OPLS-DA model.

Although significant variance was observed in the stable isotope and elemental profiles of *Zanthoxylum schinifolium* Sieb. et Zucc. across different origins, distinguishing all geographical sources reliably using any single or pairwise variable remained challenging. To identify robust markers for origin discrimination, principal component analysis (PCA) was first applied to evaluate the potential of combining multiple elements and stable isotopes for differentiating samples from the three regions. The suitability of the data for PCA was confirmed by a Kaiser–Meyer–Olkin (KMO) measure of 0.720 (KMO > 0.5). As summarized in Table S1, the first three principal components collectively explained 51% of the total variance. The PCA score plot (Figure 2A) yielded model parameters of R^2^X = 0.377 and Q^2^ (cum) = 0.139, indicating that 20.8% of the variation could be explained and 16.9% predicted by the model. As illustrated in Figure 2A, however, samples from different origins showed substantial overlap in the PCA score space. Consequently, PCA alone proved insufficient for clear geographical discrimination, highlighting the need to employ supervised classification methods such as OPLS-DA or LDA for further analysis.

OPLS-DA model was subsequently applied to distinguish the geographical origin of the samples. The training dataset, comprising 125 samples × 24 variables (four stable isotopes and 20 elements), was used to construct the model. External validation was performed on an independent set of 21 samples × 20 variables to evaluate model performance (Figure 2B). The model exhibited strong predictive and explanatory power, with a cumulative predictive ability (Q^2^Y (cum)) of 0.709 and a cumulative explained variation (R^2^Y (cum)) of 1. In the classification results, only four samples from SC were misclassified as CQ, while all other samples were correctly assigned, yielding an overall accuracy of 96.8%. VIP analysis identified the most influential discriminators, which included: Ca, δ^13^C, Mg, δ^2^H, B, δ^18^O, Cr, Ni, Na, Pb, As, Co, Se, and Zn (Figure 2C). To assess potential overfitting, a permutation test was conducted with 200 iterations. The resulting y-intercepts for R^2^ (0.0617) and Q^2^ (−0.207) were both below the respective thresholds of 0.40 and 0.05 (Figure 2D), confirming that the model is valid and not overfitted [46]. Similar OPLS-DA approaches have been successfully used in previous isotope and multi-element studies for geographical authentication of products such as peach and milk [16,47].

**Figure 3:**
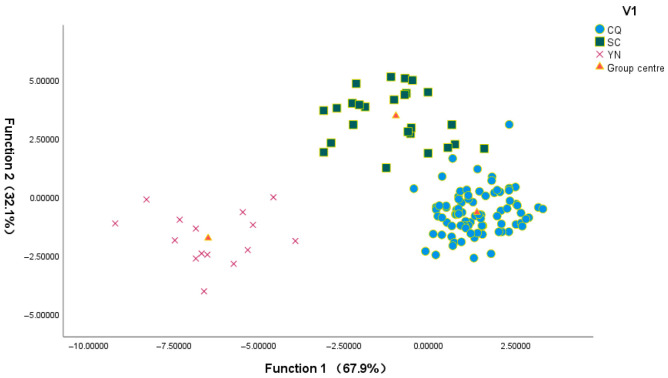
Scattering points of the first two typical discriminant functions of *Zanthoxylum schinifolium* Sieb. et Zucc. samples from different regions by LDA.

To assess how different data types influenced the classification performance for *Zanthoxylum schinifolium* origins, linear discriminant analysis (LDA) was applied separately to three datasets: stable isotope ratios alone, mineral element concentrations alone, and their combination. The resulting classification outcomes are presented in Table 4. When only stable isotope data were used, the model correctly classified 87.2% of the samples in the original validation and 84.0% in cross-validation (Table 4A). In comparison, exclusive use of mineral element data yielded higher accuracy, with 95.2% correct classification in the original validation and 88.8% in cross-validation (Table 4B). Remarkably, integrating both stable isotope and elemental profiles further improved discrimination performance, achieving 98.4% accuracy in original validation and 92.8% in cross-validation (Table 4C). Figure 3 illustrates the scatter plot of samples from the three regions. Based on Wilks’ lambda, two discriminant functions were derived, jointly accounting for 100% of the total variance (Table S4). Function 1 explained 67.9% of the variance, while Function 2 explained the remaining 32.1%. The Wilks’ lambda values for Function 1 and Function 2 were 0.033 and 0.246, with corresponding *p*-values of 1.94 × 10^−51^ and 4.61 × 10^−21^, respectively. Collectively, these results demonstrate that LDA based on the combined chemical dataset offers robust and accurate discrimination of the geographical origin of *Zanthoxylum schinifolium* across the three regions.

## 4. Conclusions

In the present study, a total of 125 *Zanthoxylum schinifolium* Sieb. et Zucc. samples were collected from three geographical origins and analyzed for stable isotope ratios (δ^13^C, δ^15^N, δ^2^H, δ^18^O) and 20 elemental contents. Multivariate statistical analysis demonstrated that combining stable isotopes with mineral elements provides an effective method for tracing the geographical origin of the samples. The OPLS-DA model achieved a high overall discrimination accuracy of 96.8%, while LDA based on the combined dataset correctly classified 98.4% of samples in the original validation. These results indicate that isotopic and elemental fingerprinting can reliably distinguish the origin of *Zanthoxylum schinifolium*. Future improvements could involve expanding the sampling scope to include more production regions, increasing the sample size across multiple harvest years, and incorporating additional environmental variables to further validate and generalize the established models.

## Figures and Tables

**Table 1:** Region information of *Zanthoxylum schinifolium* Sieb. et Zucc. samples. CQ = Chong Qing city; SC = Sichuan Province; YN = Yunnan Province.

Origin	Number	Longitude/° E	Latitude/° N	Average Altitude/m	Mean Annual Temperature (°C)
CQ	85	105.92 to 106.56	28.78 to 29.28	250.72	20.5
SC	25	102.30 to 106.56	27.47 to 32.58	1306.40	18.85
YN	15	103.23 to 105.06	26.98 to 28.24	1653.33	13.33

**Table 2:** Overview of the stable isotopes and mineral elements of all *Zanthoxylum schinifolium* Sieb. et Zucc. samples.

Isotopic Ratio [‰] or Elements (mg/kg)	Min	Max	Median	Mean	SE	Std. Dev	Coef. Var
δ^13^C (‰)	−30	−24.5	−28.5	−28.3	0.1	1.0	0.04
δ^2^H (‰)	−109	−24.1	−76.3	−76.4	1.0	11.3	0.15
δ^15^N (‰)	−0.918	8.41	2.57	2.75	0.13	1.40	0.51
δ^18^O (‰)	21.4	30.3	25.3	25.3	0.1	1.4	0.06
K	13,608	28,273	20,045	20,042	230	2567	0.13
Ca	4165	17,049	6237	6886	185	2074	0.30
P	2110	5468	3421	3465	50	560	0.16
Mg	1149	3787	1761	1897	42	470	0.25
Na	95	870	136	196	16	174	0.89
Mn	15.7	214	59.3	65.7	3.1	34.3	0.52
Fe	36.8	120	55.9	60.9	1.5	16.6	0.27
Sr	6.17	72.2	23.1	26.2	1.0	10.7	0.41
Ba	4.52	84.7	20.6	24.0	1.2	13.1	0.54
Zn	9.62	311	15.3	21.8	3.3	36.4	1.67
Cu	4.85	18.5	7.07	8.72	0.29	3.20	0.37
Ni	0.93	19.9	5.97	6.35	0.30	3.38	0.53
B	0.06	20.3	5.385	6.30	0.34	3.79	0.60
Cr	0.007	1.62	0.303	0.428	0.031	0.348	0.81
Pb	0.144	19.5	0.368	0.757	0.214	2.389	3.16
Cd	0.043	2.81	0.246	0.279	0.030	0.335	1.20
Mo	0.0257	1.25	0.154	0.201	0.014	0.160	0.80
As	0.0288	1.35	0.0605	0.0974	0.0175	0.1962	2.01
Se	0.0059	0.0822	0.031	0.0327	0.001	0.016	0.50
Co	0.0313	0.351	0.0776	0.103	0.006	0.070	0.68

Notes: Median, median values from the four regions; mean, average values from the three regions; SE, Standard error; Std. Dev, standard deviation; Coef. Var, coefficient of variation. The coefficient of variation (CV) measures the degree of variation within an element (less than 0.2, has a low degree of variation; between 0.2 and 0.3, a medium variation; greater than 0.35, highly variable).

**Table 3:** Mean stable isotopes values, multi-element contents and standard deviations of *Zanthoxylum schinifolium* Sieb. et Zucc. samples from different provinces.

Isotopic Ratio [‰] or Elements (mg/kg)	CQ (*n* = 85)	SC (*n* = 25)	YN (*n* = 15)	*p*
δ^13^C (‰)	−28.8 ± 0.5 b	−27.3 ± 0.8 a	−26.7 ± 0.8 a	***
δ^2^H (‰)	−72.3 ± 8.6 a	−80.1 ± 10.2 b	−93.6 ± 8 c	***
δ^15^N (‰)	2.97 ± 1.46	2.25 ± 0.98	2.32 ± 1.38	ns
δ^18^O (‰)	25.1 ± 1.1 b	26.4 ± 1.2 a	24.2 ± 2 b	***
K	20,357 ± 2517	19,081 ± 2346	19,861 ± 2941	ns
Ca	5823 ± 899 b	9174 ± 1786 a	9094 ± 2517 a	***
P	3399 ± 493 b	3341 ± 418 b	4050 ± 775 a	*
Mg	1694 ± 246 c	2090 ± 481 b	2719 ± 400 a	***
Na	141 ± 38 b	257 ± 234 b	409 ± 304 a	**
Mn	70.2 ± 36.1 a	51.7 ± 23.4	63.7 ± 34.9	ns
Fe	61.5 ± 17.1	61.9 ± 18.6 a	56.1 ± 7.8	ns
Sr	26.9 ± 10.9 a	23.9 ± 8.3	25.8 ± 13	ns
Ba	25.2 ± 14.1 a	23.3 ± 11.2	18.4 ± 7.3	ns
Zn	17.7 ± 7.2 b	39.6 ± 79 a	15.1 ± 2.4 ab	**
Cu	9.15 ± 3.48 a	7.69 ± 2.34	7.94 ± 2.21	ns
Ni	7.43 ± 3.33 a	3.48 ± 1.62 b	5.01 ± 2.58 b	***
B	6.36 ± 3.52 b	8.91 ± 2.77 a	1.3 ± 1.35 c	***
Cr	0.544 ± 0.353 a	0.194 ± 0.135 b	0.126 ± 0.103 b	***
Pb	0.396 ± 0.201	2.208 ± 5.158 a	0.381 ± 0.115	ns
Cd	0.239 ± 0.094	0.446 ± 0.711 a	0.23 ± 0.104	ns
Mo	0.155 ± 0.108 b	0.263 ± 0.101 a	0.353 ± 0.309 a	***
As	0.0624 ± 0.0215	0.23 ± 0.416 a	0.0742 ± 0.034	ns
Se	0.0378 ± 0.0143 a	0.0249 ± 0.0188 b	0.0167 ± 0.0036 b	***
Co	0.0859 ± 0.0514 b	0.101 ± 0.047 b	0.201 ± 0.106 a	***

Values are expressed as the mean ± SD. Different superscript letters (a–c) in each row designate significant differences (*p* < 0.05). ns: *p*-value > 0.05, *: 0.05 > *p*-value > 0.01, **: 0.01 > *p*-value > 0.001, ***: *p*-value ≤ 0.001.

**Table 4:** Classification of *Zanthoxylum schinifolium* Sieb. et Zucc. samples via LDA.

Geographical Origin	Predicted Group (Original/Cross-Validated)			Correctly Classified% (Original/Cross-Validated)
	CQ	SC	YN	
(**A**). Stable isotopes				
CQ (85)	82/82	3/3	0/0	96.5/96.5
SC (25)	6/7	17/16	2/2	68.0/64.0
YN (15)	0/2	5/6	10/7	66.7/46.7
				87.2/84.0
(**B**). Element profiles				
CQ (85)	83/80	2/5	0/0	97.6/94.1
SC (25)	4/7	21/18	0/0	84.0/72.0
YN (15)	0/2	0/0	15/13	100.0/86.7
				95.2/88.8
(**C**). Stable isotopes and elements combined				
CQ (85)	84/82	1/3	0/0	98/96.5
SC (25)	1/5	24/20	0/0	96/80
YN (15)	0/0	0/1	15/14	100/93.3
Total				98.4/92.8

## Data Availability

The original contributions presented in this study are included in the article/Supplementary Materials. Further inquiries can be directed to the corresponding author.

## Supplementary Materials

The following supporting information can be downloaded at: https://www.mdpi.com/article/10.3390/foods15061088/s1, Table S1: Principal component analysis of the first three-axis eigenvalues and variance interpretation rate.; Table S2: The method limit-of-detection values of all analyzed elements; Table S3: Element concentration of three standard materials; Table S4: Discriminant functions elaborated based on the combination of stable isotope ratios and element contents; Figure S1: Hotelling’s T^2^(A) and DModX(B) control charts for PCA outlier diagnostics.
