# Supplementary material for: Geographical Traceability of Zanthoxylum schinifolium Sieb. et Zucc. Using Stable Isotope and Multi-Element Fingerprinting Combined with Chemometrics
Source: Foods. 2026 Mar 20;15(6):1088. doi: 10.3390/foods15061088 (PMC13026058; doi:10.3390/foods15061088)
Supplement: Supplementary file 1 [file foods-4178287-supplementary.doc.author revise2.0.pdf › foods-4178287-supplementary.doc.author revise2.0.pdf]

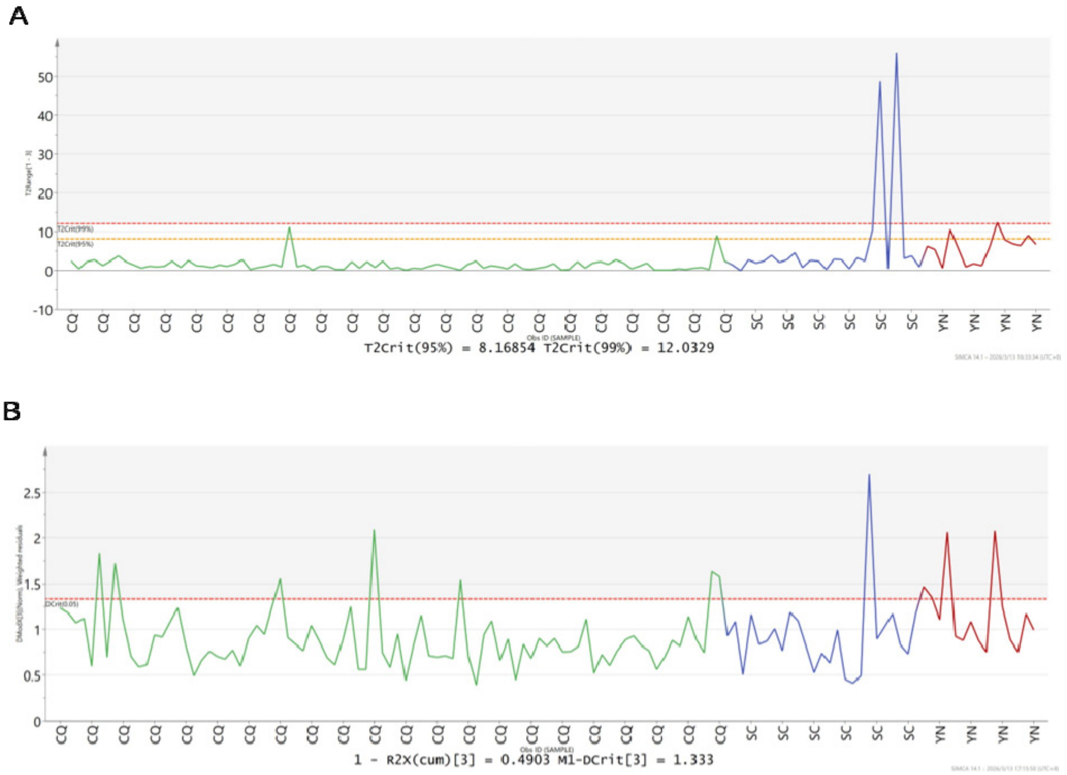

Figure S1. Hotelling's T^2(A) and DModX(B) control charts for PCA outlier diagnostics.

(A) Hotelling's T^2 statistic, which measures each sample's distance from the origin within the principal component subspace. The orange dashed line indicates the 95% confidence limit, and the red dashed line indicates the 99% confidence limit. (B) DModX, which measures the residual variance of each sample not explained by the PCA model. The red dashed line represents the critical distance (D-Crit) at the 0.05 significance level. Samples exceeding these thresholds are considered statistical outliers relative to the PCA model. Consistent with the exploratory objective of this study, all samples were retained in the final analysis to preserve the full dataset structure and avoid introducing selection bias.
